# Acute oral toxicity assessment of ethanolic extracts of *Antidesma bunius* (L.) Spreng fruits in mice

**DOI:** 10.1016/j.toxrep.2021.06.010

**Published:** 2021-06-17

**Authors:** Maria Nilda M. Muñoz, Urdujah G. Alvarado, Jerica Isabel L. Reyes, Kozo Watanabe

**Affiliations:** a Research & Development Extension, Cagayan State University, Tuguegarao City, Philippines; b Center for Natural Sciences and Environmental Research (CENSER), De La Salle University, Metro Manila, Philippines; c Graduate School of Science and Engineering, Ehime University, Japan; d Center for Marine Environmental Studies (CMES), Ehime University, Japan

**Keywords:** Acute toxicity, *Antidesma bunius* (L.) Spreng, Mice, Blood chemistry, GC–MS, Phytochemicals

## Abstract

The aim of the study is to assess the acute oral toxicity of ethanolic extracts of *Antidesma bunius* (L.) Spreng [Bignay] in ICR mice in accordance to OECD guideline 423. Single doses of Bignay fruit extracts ranging from 500 mg/kg to 2000 mg/kg, as well as a vehicle control, were given orally and monitored for 14 days. We observed that there is no mortality or adverse effects after treatment of mice indicating that Bignay extracts are safe for use in laboratory animals. Behavioral, respiratory, and neurologic changes, as well as changes in body weight, food and water consumption, did not occur during the experimental period. Hematological- (total-red and -white blood cells) and biochemical-profile analysis (alanine transaminase, blood urea nitrogen, and creatinine) remained within normal concentrations in treated mice regardless of sex. The morphology of visceral organs in all treated mice was typical in appearance when stained with H&E. It is assumed that the LD_50_ is greater than 2000 mg/kg and there is no mortality at the maximum dose used (2000 mg/kg). We have also determined the active components of dried Bignay by headspace GC–MS and by phytochemical analysis of ethanolic Bignay extracts. We have identified 16 compounds by GC–MS and 10 secondary metabolites. In this study, the assessment of extracted *Antidesma bunius* (L.) Spreng fruits [Bignay] on the value of safety measures in mice is described.

## Highlights

• In treated mice, mortality during 14-day experimental period was not observed.
• Bignay extract did not cause behavioral, respiratory and neurologic changes.
• Liver, kidney, stomach, intestines and esophagus remained intact post Bignay treatment.
• 16 volatile compounds and 10 secondary metabolites were identified.

## Graphical abstract

**Figure:**
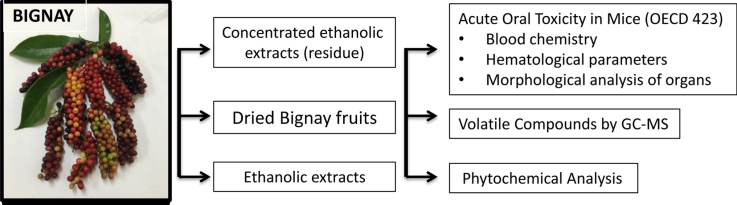

## 1 Introduction

The regulatory authorities of WHO (World Health Organization) are now more concerned with the safety and efficacy of plant-derived medicines during usage [1]. It has been reported that some plants do not only contain toxic secondary metabolites but are also contaminated with air pollutants especially heavy metals that could result to serious health problems [2,3]. Thus, there is a need to assess the safety of plant extracts for human consumption prior considering its potential therapeutic role. One of the effective ways by which this can be done is through conducting acute oral toxicity tests *in vivo.*

Acute toxicity is characterized by unfavorable effects that occurs either immediately or within a specific time frame following the administration of a single or multiple doses of a substance [[4], [5], [6], [7]]. Any effect that causes impairments in organs and/or biochemical lesions, which in turn perturbs the functioning of the body in general or individual organs, is referred to as an unintended (or adverse) effect. Acute and sub-acute toxicity tests are regularly conducted for examination of natural products or synthetic drugs [[4], [5], [6], [7]]. This acute toxicity testing is the first step in determining the effects of new investigational drugs within 14 days of a single or multiple dose administration. It is normally given orally to test animals, usually rat or mice, to assess the median lethal dose (LD_50_) for a specific toxic drug. The LD_50_, the dose that kills 50 % of the research animal population, is now used as a significant parameter in calculating acute toxicity as well as an initial procedure for general toxicity screening of chemical and pharmacological agents. Other biological effects, as well as the time of onset, period, and degree of recovery on survived animals, are also important in acute oral toxicity evaluation.

*Antidesma bunius* (L.) Spreng, known as “Bignay” in the Philippines, and cultivated in parts of Northeast Thailand and India has started to become an interest in numerous studies because of its promising potentials [[8], [9], [10], [11], [12], [13], [14], [15], [16]]. Ripe Bignay fruits can be eaten raw just like blueberries, raspberries, cherries and strawberries. Others used the fruits as food flavors, fruit preserves and variety of drinks [[16], [17], [18]].

Previous studies have reported that Bignay demonstrated anti-bacterial [13], anti-oxidant [18,19], α-glucosidase inhibitory activity [12,20] and anti-cancer [8] properties. Although Bignay has been reported to contain bioactive compounds [15,21] for treatment of various ailments, little is known about the safety and toxicity health issues of these fruits [10,22]. Studies *in vivo* reported that ethanolic Bignay seed extracts at 250 mg/kg revealed potential renal and hepatic adverse effects as seen in both normal and diabetic rats, evident in abnormal blood levels of creatinine, albumin and alkaline phosphatase [10]. In the same study, the acute oral toxicity effects showed absence of deformities in critical organs including kidney and liver at maximum concentration of 2000 mg/kg [10]. However, studies using whole Bignay fruit extracts are yet to be investigated.

It is important to ensure that the medicinal plants are genuine. Taxonomists used DNA barcoding as a biodiversity exploration method because it produces repeatable results [23]. Bignay is a common plant in the Philippines, and professional taxonomists have verified the plant specimens (National Museum Manila; Botany Division, control #17-06954 DNT). To date, assessment of acute oral toxicity of Bignay fruits has not been fully examined in mice. The aim of the study is to determine the acute oral toxicity of ethanolic extracts of *Antidesma bunius* (L.) Spreng [Bignay] in ICR mice in accordance with OECD guideline 423. This *in vivo* technique will demonstrate a repeatable method for measuring acute toxicity that, if implemented, could lead in more consistent and reproducible results.

## 2 Materials and methods

### 2.1 Plant materials

*Antidesma bunius* (L.) Spreng [Bignay] fruits were harvested from Tabuk Farm located in Cagayan Valley Region 2, Philippines between May-July 2019, and authenticated by the National Museum, Philippines (Botany Division, Control # 17-06-954 DNT). It is a good practice to collect Bignay fruits from a single source so that data is reliable and heavy metals and other components are not retested. According to preliminary results, heavy metals are undetectable in the soil, so it is assumed that the fruits have not absorbed any elements from the soil. The freshly harvested fruits were washed with tap water, rinsed with distilled water and dried in oven at 50 °C for 4 days or until the % moisture content is 13 %.

### 2.2 Preparation of ethanolic extracts of Bignay for different assays

Bignay fruits were dried, pulverized, and 70 % laboratory grade ethanol (Sigma-Aldrich Pte. Ltd, Philippines) was used to extract it in a 10:30 (g:mL) ratio. During the day, the extracted fruits were kept at 50−60 °C with regular stirring. The mixture was stored at 4 °C overnight, then removed from the refrigerator and agitated for another 2 h at room temperature before gravity filtration with Whatman filter paper #1 (Sigma-Aldrich Co.). To extract the residual ethanol, the filtrate was concentrated under vacuum using a rotary evaporator (Fisher Scientific) set at 50 °C. The residue containing complete Bignay components was resuspended in distilled water and stored at 4 °C in an airtight container before use.

Secondary metabolites were measured using colorimetric analysis: alkaloids, steroids, saponins, flavonoids, tannins, coumarin, and other compounds were determined using ethanolic Bignay extracts [24]. For dosing animal, fruit extracts were concentrated using rotary evaporation. The residue, which is made up of a mixture of secondary metabolites, was resuspended in distilled water to get the right concentration for the 500−2000 mg/kg dose needed to treat the animals. According to Guidance Document on Acute Oral Toxicity Testing: “The standard pre-specified fixed doses are 5, 50, 300 or 2000 mg/kg. There is an option to use an additional dose level of 5000 mg/kg, but only when justified by a specific regulatory need”.

Groups of animals are dosed in a stepwise procedure, with the initial dose being selected as the dose expected to produce mortality in some animals. Further groups of animals may be dosed at higher or lower fixed doses, depending on the presence of mortality, until the study objective is achieved; that is, the classification of the test substance based on the identification of the dose(s) causing mortality, except when there are no effects at the highest fixed dose.

### 2.3 Heavy metals

The analysis of heavy metals was analyzed using Atomic Absorption Spectroscopy (Thermo Fisher Scientific). Lead (Pb) and Cadmium (Cd) were measured from 3 separate extractions of Bignay fruits using the absorption of optical radiation by free atoms in the gaseous state [2]. Data are expressed in parts per million (ppm).

### 2.4 Preparation of experimental animals

The ICR (Institute of Cancer Research) mice were purchased from Research Institute of Tropical Medicine, Alabang, Muntinlupa, Philippines. All animals of either sex (8–10 weeks old) were housed in Animal Research Facility, and were randomly marked on the tail for proper identification. The male and female animals were separated after marking to prevent mating. Standard procedures were performed maintaining the mice on a 12-h light/dark cycle at specific room conditions for a week to acclimatize the mice prior to experimental procedures [25]. All animals had regular supply of drinking water and food. The experimental protocol was reviewed and approved by the Institutional Animal Care and Use Committee of the College of Veterinary Medicine, University of the Philippines, Los Baños, Laguna. Guidelines from Bureau of Animal Industry, Philippines was issued and followed for proper care and use of experimental animals.

### 2.5 Acute oral toxicity testing (OECD 423)

The acute oral toxicity test was conducted in 20 male and 20 female ICR (8–10 weeks old) mice according to the procedures outlined by the Organization for Economic Co-operation and Development (OECD) 423 [26,22]. The animals were randomly selected and grouped into untreated (receiving distilled water only) and treated (receiving Bignay fruit extract) set. Following overnight fasting period, the animals were weighed and single doses of Bignay fruit extracts ranging from 500 mg/kg to 2000 mg/kg, as well as a vehicle regulation, were given orally and monitored for 14 days.

The 4 treatment groups (n = 5 male per group; n = 5 female per group) were as follows:

a Group 1: distilled water, serves as vehicle control (untreated)
b Group 2: 500 mg/kg Bignay fruit extracts
c Group 3: 1000 mg/kg Bignay fruit extracts
d Group 4: 2000 mg/kg Bignay fruit extracts

After 60 min, all of the animals were given food and water. The treated mice were then monitored for 30 min after dosing, then hourly for 8 h, and everyday for the next 14 days. For each treated animal, all findings were recorded. Throughout the experiment, all animals were weighed, and visual observations for death, behavioral patterns (moving around, drowsiness, seizures), physical appearance changes (inability to move, body shaking), and injury were made. Throughout the 14-day testing period, food and water intake (volume) were also assessed daily. To calculate the food intake, the remaining feeds were weighed and subtracted from the initial weight. Water intake was also reported for each treatment group.

One drop of eye anesthetic tetracaine (Sigma-Aldrich Co., Philippines) was applied to each mouse's right eye prior to blood collection. At Day 1 and Day 14, 250 μL blood was obtained from the retro-orbital sinus two minutes apart using heparinized micro-hematocrit tubes. Using an automated blood chemistry analyzer, the total red and white blood cell counts were measured, and the blood serum was used to calculate alanine transaminase (ALT), blood urea nitrogen (BUN), and creatinine (CREA) (IDEXX, USA).

Mice were sacrificed by intraperitoneal injection of pentobarbital sodium (Sigma-Aldrich Chem., Philippines) at a dose of 80 mg/kg body weight at the end of the experiment. The liver, kidneys, and other visceral organs were removed and investigated for any abnormalities on a gross level.

Organs were weighed and then fixed in a 4 percent paraformaldehyde solution for at least 72 h before being processed with the standard paraffin technique. For microscopic inspection at 40X magnification, the fixed organs were sectioned at 5 μm thickness and stained with Hematoxylin and Eosin dye (H&E; Sigma-Aldrich Co., Philippines).

### 2.6 Identification of compounds

#### 2.6.1 Gas chromatography analysis

This experiment used a headspace Gas Chromatography-Mass Spectrometry (GC–MS; Shimadzu Corp., Japan) apparatus to distinguish the chemical compounds in dried and pulverized *Antidesma bunius* (L.) Spreng fruits. Supelco® 24 gauge 100 m Polydimethylsiloxane (PDMS) Solid Phase Microextraction (SPME) fiber was used in this experiment. Other fiber coating was also used such as 50/30 μm 24-gauge Divinylbenzene/Carboxen/Polydimtheylsiloxane (DVB/CAR/PDMS) SPME Fiber if needed throughout the analysis. SPME fibers were baked prior to use according to manufacturer’s instructions. A 500 mL Pyrex® Erlenmeyer flask coated with aluminum foil and parafilm was used as a headspace chamber for the headspace review. Before use, flasks were washed with technical grade acetone and baked for 1 h at 150 °C. After cooling, each sample was put in separate clean flasks and heated to 30 °C–40 °C for 25 min while the PDMS fiber was exposed. After incubation, SPME fiber was immediately injected into the GC–MS. Blank trials of the GC–MS, SPME fiber, and flasks were also carried out in order to eliminate any potential errors during the study. The Shimadzu GC–MS QP2020 was used in this analysis, with a SH-Rxi-5Sil capillary column (30 m x0.25 mm ID x 0.25 m df). The injector port was manually injected with SPME fiber. A 0.75 mm ID Shimadzu® SPME Inlet Liner and a Restek® Merlin Microseal Septa General Purpose Kit were installed in the injector port. MS Settings were set to SCAN Mode (35 *m/z* to 500 *m/z*) and acquisition mode to SCAN Mode (35 *m/z* to 500 *m/z*). For library matching, libraries from the NIST 2017 Mass Spectral Library and the Wiley Registry 11th edition were used. The temperature program was set to start at 50 °C for 5 min, then increase by 10 °C every 10 min until it reached 200 °C. Desorption of volatiles was carried out at 250 °C in split less mode with Linde® Helium as the carrier gas at a rate of 1 mL/min (99.995 %). The relative quantity of chemical compounds present in each of the extracts of Bignay fruit was expressed as a percentage based on the peak area generated in the chromatogram. Trials were carried out in threes.

#### 2.6.2 Phytochemical analysis: Qualitative determination of secondary metabolites

Secondary metabolites were detected directly using an ethanolic extract of *Antidesma bunius* (L.) Spreng fruits. In three runs from three different collected samples, at least ten secondary metabolites were measured qualitatively [24].

##### 2.6.2.1 Test for alkaloids (Mayer’s test)

To a total of 0.5 mL of ethanolic fruit extract, 2.5 mL 2% hydrochloric acid (HCl) solution was added followed by 0.25 g powdered NaCl. The mixtures were then stirred and filtered. The residues obtained were washed with 2% HCl solution and 2.5 mL distilled water was added to the residue. To 0.5 mL of mixture, 1 mL of Mayer’s reagent was added. The presence of turbidity indicates the presence of alkaloids.

##### 2.6.2.2 Test for combined anthraquinones

A total of 0.5 mL of ethanolic fruit extract was boiled with 1 mL of 10 % HCl for 5 min. While still hot, the mixture was filtered and allowed to cool followed by addition of equal amount of chloroform (CCl_4_). The CCl_4_ layer was aspirated into a clean dry test tube using a pipette. Equal volume of 10 % NH_4_Cl solution was added into the CCL_4_ containing test tube. The mixture was agitated and allowed to separate. The separated aqueous layer was monitored for any color change; delicate rose pink color indicates the presence of anthraquinones.

##### 2.6.2.3 Test for carotenoids

To 0.5 mL of ethanolic fruit extract, 2.5 mL of CCl_4_ was added. The mixtures were vortexed for 1 min and were filtered using Whatman filter paper #1 (Sigma-Aldrich Chemical Co.). To the filtrate, 1.5 mL 85 % sulfuric acid (H_2_SO_4_) was added. Formation of a blue color at the interface indicates the presence of carotenoids.

##### 2.6.2.4 Test for coumarins

To 0.5 mL of the ethanolic fruit extract, 0.5 mL of 10 % sodium hydroxide (NaOH) was added to the mixture. Formation of yellow color indicates the presence of coumarins.

##### 2.6.2.5 Test for flavonoids

To 0.5 mL of the ethanolic fruit extract, distilled water was added to a final volume of 2.5 mL and was boiled for 5 min. The sample was filtered, and a few drop of 20 % NaOH solution was added to the filtrate. A change of color yellow to colorless indicates the presence of flavonoids after addition of 10 % HCl.

##### 2.6.2.6 Test for saponins

Extracted fruit sample (1 mL) was diluted in 20 mL distilled water. The mixture was shaken in a graduated cylinder for 15 min. Development of stable foam suggests the presence of saponins.

##### 2.6.2.7 Test for steroids

A total of 0.5 mL ethanolic fruit extract was mixed in a 2.5 mL of CCl_4_. Equal volume of concentrated H_2_SO_4_ was then added carefully through the side of the test tube. The presence of steroids showed when upper layer turned red and the sulfuric acid layer showed yellow with green fluorescence.

##### 2.6.2.8 Test for tannins

A 0.5 mL ethanolic fruit extract is boiled in equal amount of distilled water and was filtered prior to addition of 0.2 mL 0.1 % ferric chloride (FeCl_3_). A brownish green or blue-black coloration shows the presence of tannins.

##### 2.6.2.9 Test for terpenoids

A 0.25 mL CCl_4_ was added to 0.5 mL ethanolic fruit extract and gradually; 1 mL of concentrated sulfuric H_2_SO_4_ was added to form a layer. A reddish brown precipitate coloration at the interface formed indicates the presence of terpenoids.

##### 2.6.2.10 Test for quinones

One mL of 70 % laboratory grade ethanol was added to 0.5 mL ethanolic fruit extract followed by addition of 1 mL potassium hydroxide (KOH) to the mixture. Formation of blue color indicates the presence of Quinone.

### 2.7 Statistical analysis

All data are expressed as mean ± standard error (SE) of the mean. Since there was no difference in data between vehicle control and treated mice for all parameters, statistical analysis was not necessary and was not carried out in this study.

## 3 Results

### 3.1 Heavy metals

Cadmium (Cd) and lead (Pb) were found in small amounts in Bignay extract. Cd levels were 0.002 ppm in Bignay, while Pb levels were 0.5033 ± 01,354 ppm. Three times the samples were analyzed, and the results were published in parts per million (ppm). According to the findings, the laboratory mice are unlikely to be affected by a small amount of heavy metals [2,3].

### 3.2 Acute oral toxicity study

Both animals given the highest oral dose of Bignay, 2000 mg/kg, survived the entire experimental period and displayed no signs of discomfort after dosing or for up to 14 days afterward. When taken as a whole, Bignay's median lethal dose (LD_50_) is greater than 2000 mg/kg body weight.

### 3.3 Physiological parameters

The composite mean body weight of male and female mice given distilled water (SI Table 1/2), 500 mg/kg, 1000 mg/kg, or 2000 mg/kg of Bignay fruit extracts (BFE) is shown in Table 1. Following administration of BFE, there were no major variations in total body weight (Table 1) or weight gain (Table 2). The animals were given samples of a concentrated ethanolic BFE, and the resuspended residue in distilled water, which included all of Bignay's total components, was used to treat them.

**Table 1:** Mean Body Weight of Treated Male and Female Mice after 14-day experimental period. There is no statistical difference when male body weight is compared to female group. Bignay Fruit Extracts (BFE).

Treatment Groups	Mean Body Weight of Male (g; n = 5 mice)	Mean Body Weight of Female (g; n = 5 mice)
Distilled water	31.16 ± 0.90	30.81 ± 0.68
500 mg/kg BFE	31.40 ± 0.90	31.14 ± 0.94
1000 mg/kg BFE	31.11 ± 0.99	30.68 ± 1.03
2000 mg/kg BFE	31.40 ± 1.03	31.64 ± 0.85

**Table 2:** Mean Body Weight Gain of Male and Female Mice during the testing period regardless of treatment. There is no difference in body gain between male (n = 5 mice) and female group (n = 5 mice). Bignay Fruit Extracts (BFE).

Treatment Groups	Mean Body Gain of Male (g)	Mean Body Gain of Female (g)
Distilled water	2.25 ± 0.06	2.22 ± 0.05
500 mg/kg BFE	2.24 ± 0.06	2.20 ± 0.06
1000 mg/kg BFE	2.22 ± 0.07	2.19 ± 0.07
2000 mg/kg BFE	2.24 ± 0.07	2.26 ± 0.07

The normal feeds and water intake of treated mice were also assessed. SI Table 2 indicates the average daily feed and water intake of male and female mice in each treatment group (SI Table 3). With the exception of sex and weight, the feeds ingested by treated and untreated mice were within the usual range of 10 g/100 g body weight/day/mouse. Water intake was also comparable, falling within the usual range of 10 mL/100 g body weight/day/mouse. There was no substantial difference in the weight of visceral organs excised from male mice (SI Table 4) or female mice (SI Table 5) treated with distilled water or mice given increasing doses of Bignay. Our findings showed that oral administration of Bignay fruit extracts had no effect on the metabolism of treated mice or caused any damage to their visceral organs over the course of the study.

After receiving distilled water or increasing doses of Bignay fruit extracts, none of the animals showed any physiological improvements. All treated mice, regardless of age, sex, or weight, showed no apparent changes in behavior (moving around, anxious, inability to walk), nervous reaction (seizures, drowsiness), or respiratory signs (hard to breathe, mucus secretion).

### 3.4 Hematological examination

Hematological examination was performed on blood samples obtained in tubes containing anticoagulant. Table 3 shows the rate of hematological parameters in male and female mice. We showed that the number of tRBC and tWBC measured on Day 1 and Day 14 for all treated groups was comparable. The usual limits for tRBC and tWBC were 7.9−10 × 10^6^/uL and 4000−11,000/uL, respectively [27].

**Table 3:** Total Red Blood Cell (tRBC) and White Blood Cell (tWBC) count at Day 1 and Day 14 for all treated groups. (n = 10 mice). Oral administration of Bignay extract did not change the numbers of tRBC and tWBC during the treatment period. Bignay Fruit Extracts (BFE).

Treatment Groups	tRBC Count Female x 10^6^/μL		tRBC count Male x 10^6^/μL		tWBC count Female		tWBC count Female	
	Day 1	Day 14	Day 1	Day 14	Day 1	Day 14	Day 1	Day 14
Distilled Water	7.77 ± 0.03	7.83 ± 0.02	8.11 ± 0.55	7.99 ± 0.38	4,703 ± 63.0	4,733 ± 82.2	4,763 ± 74.4	4,788 ± 64.2
500 mg/kg BFE	7.70 ± 0.57	7.62 ± 0.38	7.73 ± 0.31	7.78 ± 0.17	4,780 ± 99.2	4,791 ± 83.4	4,781 ± 50.5	4,775 ± 72.3
1000 mg/kg BFE	7.75 ± 0.32	7.77 ± 0.18	7.8 ± 0.05	7.8 ± 0.23	4,800 ± 87.2	4,814 ± 62.3	4,805 ± 100.0	4,806 ± 95.1
2000 mg/kg BFE	7.88 ± 0.37	7.93 ± 0.24	7.9 ± 0.17	7.9 ± 0.15	4,751 ± 59.9	4,812 ± 43.4	4,819 ± 74.6	4,838 ± 94.1

### 3.5 Blood chemistry

For the serum biochemistry profile analysis, blood samples without anticoagulant were used. Table 4 shows the mean ALT, BUN, and CREA levels measured on Days 1 and 14. There was no difference in serum ALT, BUN, or CREA levels between the treated and control groups. Our findings indicate that the Bignay fruit extracts did not cause any liver or kidney damage.

**Table 4:** Mean blood alanine transaminase (ALT), blood urea nitrogen (BUN), and creatinine (CREA) in treated male and female mice. All values are expressed as mean ± SEM. Bignay Fruit Extracts (BFE).

	Male (n = 5 mice)						Female (n = 5 mice)					
	ALT (U/L)		BUN (mg/dL)		CREA (mg/dL)		ALT (U/L)		BUN (mg/dL)		CREA (mg/dL)	
	Day 1	Day 14	Day 1	Day 14	Day 1	Day 14	Day 1	Day 14	Day 1	Day 14	Day 1	Day 14
Distilled Water	51.8 ± 5.2	49.8 ± 3.1	24.2 ± 2.6	25.5 ± 1.9	0.61 ± 0.1	0.61 ± 0.1	48.6 ± 2.1	51.4 ± 2.4	23.8 ± 3.3	25.0 ± 2.3	0.56 ± 0.07	0.58 ± 0.06
500 mg/kg BFE	49.6 ± 3.1	52.1 ± 3.25	22.0 ± 2.1	23.3 ± 3.1	0.60 ± 0.1	0.62 ± 0.07	50.0 ± 1.0	52.4 ± 2.4	22.4 ± 2.3	23.7 ± 0.06	0.59 ± 0.07	0.63 ± 0.07
1000 mg/kg BFE	49.3 ± 2.7	52.6 ± 2.7	23.8 ±2.6	25.5 ± 1.3	0.56 ± 0.2	0.59 ± 0.04	51.2 ± 2.1	54.3 ± 3.4	23.8 ± 2.1	24.0 ± 0.82	0.60 ± 0.07	0.58 ± 0.04
2000 mg/kg BFE	50.2 ± 1.9	52.6 ± 3.9	25.0 ± 4.3	26.7 ± 1.9	0.56 ± 0.3	0.57 ± 0.04	52.0 ± 5.3	53.6 ± 4.4	22.6 ± 2.4	24.5 ± 3.11	0.55 ± 0.01	0.59 ± 0.05

### 3.6 Histopathological examination

**Fig. 1:**
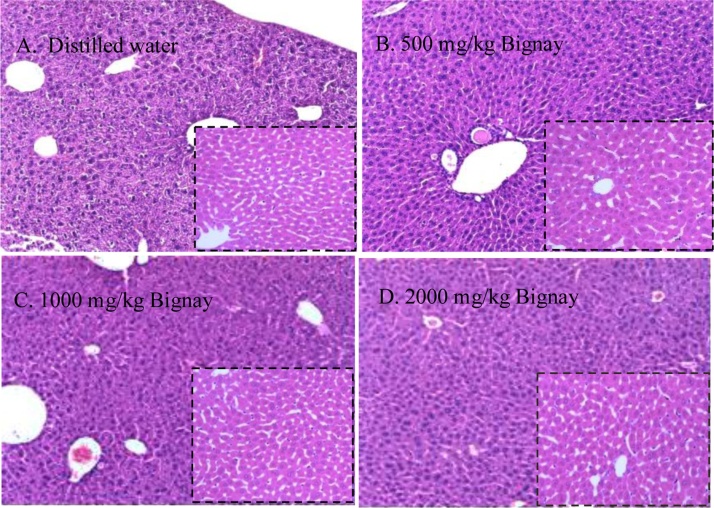
Representative photos of liver (A–D) obtained from male (*Outsized*) and female (*Inset*) mice treated with A. Distilled water; B. 500 mg/kg Bignay fruit extracts; C. 1000 mg/kg Bignay fruit extracts; and D. 2000 mg/kg Bignay fruit extracts. The liver showed normal anatomical appearance, and no sign of bleeding in all treatment groups.

The stained microsection organs presented in a blinded manner were assessed and examined by a licensed Veterinary Medical Pathologist. Prior to the evaluation and analysis of stained tissues, the evaluators were unaware of any of the treatments. The liver and kidneys are essential organs that are the primary metabolic targets of any toxic drug. Fig. 1 shows representative photomicrographs of liver stained with Hematoxylin and Eosin (H&E) dye (A–D). On macroscopic inspection of the tissue, neither laceration nor hemorrhage of the liver was observed in any of the classes. Male mice's liver was depicted in the outsized images, while female mice's liver was depicted in the insets.

**Fig. 2:**
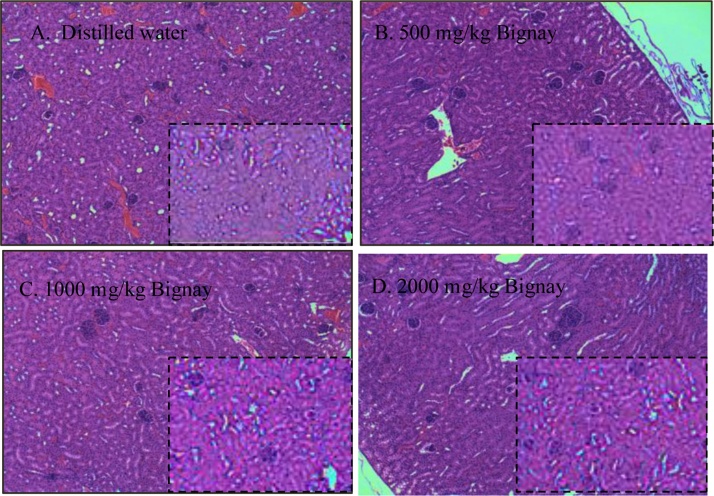
Representative photos of kidney (A–D) obtained from male (*Outsized*) and female (*Inset*) mice treated with A. Distilled water; B. 500 mg/kg Bignay fruit extracts; C. 1000 mg/kg Bignay fruit extracts; and D. 2000 mg/kg Bignay fruit extracts. The kidney showed normal anatomical appearance, and no signs of tissue rupture were seen in all treatment groups.

Fig. 2 shows representative photomicrographs of stained kidney (A–D). The tissues from both the male and female patients had a typical structural appearance. In H&E stained kidneys, no erosion, rupture, or tissue abnormalities were found.

**Fig. 3:**
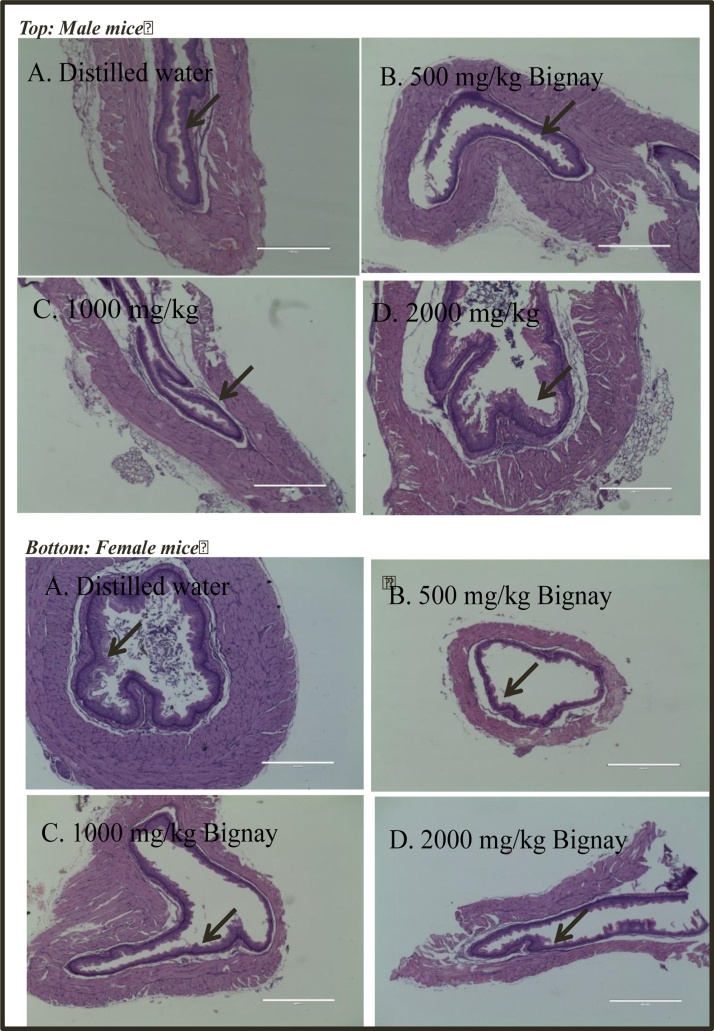
Representative photos of esophagus obtained from male (*Top*) and female (*Bottom*) treated with A. Distilled water; B. 500 mg/kg Bignay fruit extracts; C. 1000 mg/kg Bignay fruit extracts and 2000 mg/kg Bignay fruit extracts. All treatment groups were lined with stratified squamous epithelial cells (see arrow).

The usual features of the esophagus were visible in the stained microsection, which included intact epithelial cells (EPI; see arrow). In both of the handled male and female animals, there were no obvious anomalies observed (Fig. 3).

**Fig. 4:**
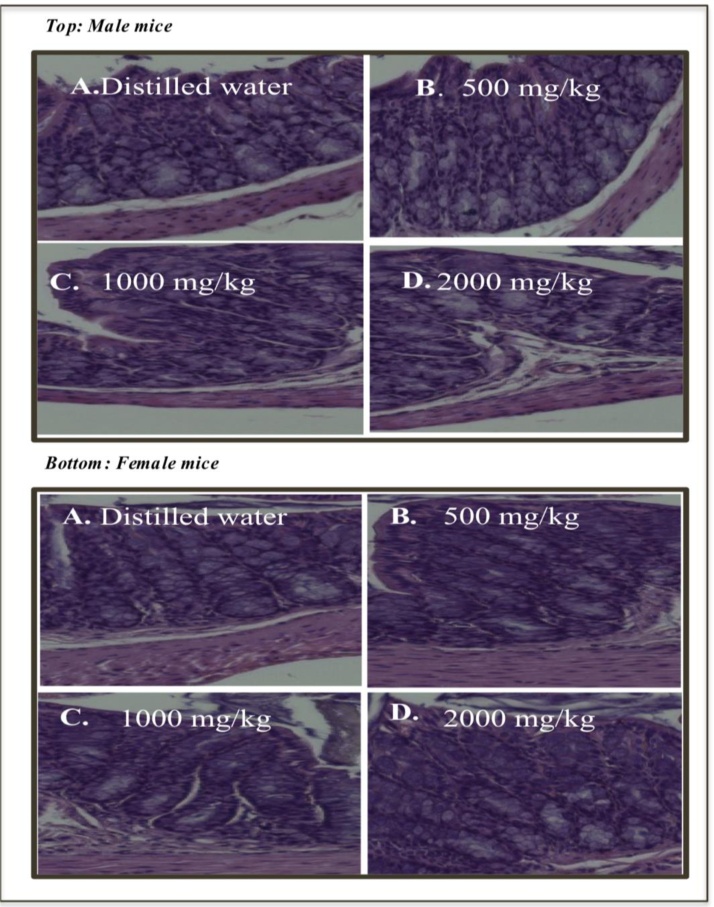
Representative photos of large intestine derived from male (Top) and female (Bottom) mice treated with A. Distilled water; B. 500 mg/kg Bignay fruit extracts; C. 1000 mg/kg Bignay fruit extracts and D. 2000 mg/kg Bignay fruit extracts (n = 10 mice). There is no significant difference in anatomical structure even at the highest dose administered orally in mice.

There were no indications of tissue damage in large intestine after treatment with either vehicle control or Bignay fruit extracts (Fig. 4). Neither signs of inflammation nor tissue erosions were observed in all treated mice regardless of sex.

### 3.7 Identification of compounds in Bignay

#### 3.7.1 GC–MS analysis

We reported the chemical profile of pulverized dried Bignay fruits using headspace gas chromatography-mass spectrometry (GC–MS; Table 5). We discovered that the major components of Bignay fruits were typical esters and aldehydes compounds, which may provide a distinct character with respect to flavor and aroma (Table 4). The major component of Bignay fruit is (E)-2-Hexenal with 31.9 % peak area. This compound is the simplest straight-chain unsaturated aldehyde of interest for perfumes and flavors. Other compound identified was d-limonene, which is widely used as a flavor and fragrance additive in cosmetics and foods because of its pleasant lemon-like odor. The compound 5,9-Undecadien-2-one, 6,10-dimethyl-,(E)- detected is similar to one of the key volatile flavor compounds of tomato [28] and certain species of mushroom [29]. Other compounds possess similar features with regard to fragrance and flavor. These are achieved on the headspace collection, a direct injection of SPME to the GC–MS. From the observed results, it is apparent that Bignay might have potential not only in management of disease but also have industrial benefits.

**Table 5:** The 16 identified compound present in *Antidesma bunius* (L.) Spreng fruits as determined by headspace GC–MS. The % area of the chromatogram is presented based on the retention time (min). The names of compounds are identified through spectral library provided in GC–MS device.

Retention Time (min)	Peak Area (%)	Name of Compound	Molecular Formula
2.16	4.19	Silanediol, dimethyl	C2H8O2Si
3.67	11.84	Hexanal	C6H12O
5.23	31.90	2-Hexanal, (E)	C6H100
5.66	1.71	Cyclohexanol	HOCH(CH2)5
5.79	2.18	1-Hexanol	CH3(CH2)5OH
9.83	0.59	D-Limonene	C10H16
12.09	0.94	(+)-2-Bornanone	C10H16O
12.06	0.95	Cyclohexanol, 5-methyl-2- (1-methyl)-,(1-alpha.,2.beta.,5.alpha.)-(.+/-)-	C10H20
12.85	1.13	Octanoic acid ethyl ester	C10H20O2
14.03	1.56	Benzoic acid,2-hydroxy-,ethyl ester	C9H10O3
15.75	0.48	Decanoic acid, ethyl ester	C12H24O2
15.85	0.61	Tetradeecane	C14H30
16.47	2.67	5,9-Undecadien-2-one,6,10-dimethly-,(E)-	C13H22O
16.63	2.07	Acenapthylene	C12H8
16.75	0.98	2-Propenoic acid, 3 phenyl-, ethyl ester, (E)	C9H8O2
18.24	1.68	3-(Isobutyriloxy)-1-isopropyl-2,2-dimethlypropyl 2methylpropanoate	C27H34O6
18.26	1.09	Dodecanoic acid ethyl ester	C13H26O2

#### 3.7.2 Qualitative analysis of secondary metabolites

Qualitative analysis showed that ethanolic extract of Bignay are rich in alkaloids, coumarins, flavonoids, saponins, steroids, tannins, terpenoids and quinones, which are medicinally important phytoconstituent. The combined anthraquinones and carotenoids were insignificant in the extracts. Table 6 is the list of secondary metabolites identified in ethanolic extracts of Bignay fruits. The scoring system is as follows: +++: highly present, ++: moderately present, +: low, −: absent.

**Table 6:** Secondary metabolites from Ethanolic Extracts of Bignay.

Secondary Metabolites		Trial 1	Trial 2	Trial 3
Alkaloids		+++	+++	+++
Comb. Anthraquinones		–	+	–
Carotenoids		–	–	+
Coumarins		++	++	+++
Flavonoids		+++	+++	+++
Saponins		+++	+++	+++
Steroids		+++	+++	++
Tannins		+++	+++	+++
Terpenoids		+++	++	+++
Quinones		+++	++	+++

## 4 Discussion

The aim of the study is to determine the acute oral toxicity of ethanolic extracts of *Antidesma bunius* (L.) Spreng [Bignay] in ICR mice, in accordance with OECD guideline 423. During a 14-day observation period, we found that single doses of Bignay fruit extracts ranging from 500 mg/kg to 2000 mg/kg caused no signs of toxicity or mortality in any of the animals studied. Accordingly, the LD_50_ is greater than 2000 mg/kg. Based on the Globally Harmonized System of Classification and Labeling of Chemicals, the substances having an LD_50_ value greater than 2000 mg/kg are considered as relatively safe. This observation is in line with the findings of [30]. However, others have reported an LD_50_ value of 3250 mg/kg body weight in rats, which was not the case in our investigation using mice. This could be attributed to differences in experimental animals and secondary metabolite compositions between plant specimens collected from various agro-ecological zones, geographical origin, genetic variances, plant section used, extraction process, maturity stage, and harvest season [31].

The current study utilized the whole fruit of *Antidesma bunius,* extracted with ethanol. Rotary evaporator removed the residual ethanol and the residue (which consists of a mixture of secondary metabolites) was resuspended with distilled water prior to oral gavage at different doses into mice. Our study is mainly focused on acute toxicity assessment of Bignay fruits in pathogen-free mice, which is easy to handle, accessible and cost-effective than rats. Mice and rats have been the preferred experimental animals for biomedical research due to their anatomical, physiological, and genetic similarity to humans [32]. Aside from rodents, zebrafish can be used to test for safety [33]. The use of zebrafish is far less expensive, and acute toxicity tests can be performed early in the preclinical research process. Zebrafish can be used to assess changes in various tissues induced by harmful effects of chemicals, medicines, or plant extracts. As such, this can be alternative *in vivo* model for acute oral toxicity testing.

We measured the heavy metals Pb and Cd because previous findings demonstrated that human exposure to such heavy metals could cause adverse health effects indicating toxic property of plants leading to neurological defects [34], renal degradation [35], bone lesions [3], and hypertension [36]. Heavy metals were significant environmental pollutants however, there were insignificant amounts of Pb and Cd measured in Bignay fruit extracts suggesting that heavy metals were not absorbed directly into the fruits.

Analysis of body weights showed no change in both the treatment and control groups, regardless of sex (Table 1). Neither weight gain in male nor in female mice was noted when data obtained from Day 1 were compared to data obtained in Day 14 (Table 2). All animals demonstrated comparable amount of feeds and water intake in male and female mice during the duration of experiments (SI Tables 2 & 3). Our findings indicated that oral administration of prepared Bignay fruit neither induces any suppression in eating habit nor had any effect on water intake.

The relative organ weight is a useful metric for determining whether or not an organ has been injured [37]. When the weight of visceral organs collected at day 14 from male (SI Table 4) and female mice (SI Table 5) was compared to the untreated category, there was no difference. These findings have revealed that Bignay is relatively healthy for mice at the doses used in the study.

Regardless of age, sex, or weight, behavioral findings after various dosing showed normal respiration rates and no improvement in body activity for the first hour. Both groups of treated mice exhibited normal behavior (no convulsions, scratching, or twisting) during the 14-day observation period, indicating that Bignay fruit had no adverse reaction in mice.

The study of blood parameters (tRBC and tWBC) is crucial in the assessment of hematological lesions, and any adjustment can often indicate hematopoietic system lesions [38]. Both blood cells are produced from pluripotent stem cells during hematopoiesis, which is the process of blood cell creation [38]. *In vivo* toxicity may cause irregular low RBC levels due to an immature production of reticulocytes and hemolysis [39]. On the other hand, elevation of WBCs, signals the activation of immune response that serves as protective mechanism against foreign substances [40]. Previous studies have demonstrated that absorption of toxic plants resulted in an alteration in the normal levels of tRBC and tWBC [41]. Fruit extracts from *Antidesma bunius* Spreng had no effect on hematological parameters, implying that the fruit extracts have no effect on bone marrow activity. The values of tRBC and tWBC cell count (Table 3) were within the published normal values recorded by Olfert from Day 1 to Day 14 [27]. This validates the fact that at all doses of Bignay fruit extracts are likely safe for curative applications.

The serum enzymes, ALT, BUN and CREA are indicative of liver and kidney damage [42,43]. The ALT is a cytoplasmic enzyme secreted by liver and increased in level may suggests hepatocellular damage. Both male and female mice had no increase in enzymes in their serum, suggesting that Bignay extracts have a liver-protective effect in treated mice (Table 4). This was further verified by histological analysis, which revealed that stained liver tissue had a regular structural appearance and no internal bleeding (Fig. 1A–D).

The BUN and CREA are measures of glomerular filtration rate, which is a measure of how well the kidneys are working [44]. Increased levels of these parameters in the blood indicate a problem with the kidneys' ability to filter the blood. In all treated mice, we found no rise in BUN and CREA levels in the serum (Table 4).

After histopathological examination, no evidence of renal injury was found (Fig. 2A–D). These results proved that Bignay fruit extracts do not cause kidney damage.

Additional histopathological evaluations were carried out to examine any damages and appearance in stomach and large intestine following treatment with Bignay fruit extracts (Figs. 3 & 4). We observed that there were no alterations in appearance of stomach (Fig. 3) and large intestine (Fig. 4) in all treated mice demonstrating that Bignay extract is simply safe for experimental use. Although Bignay fruit extract has no adverse effect in mice using acute oral toxicity test, more researches warrant further studies to validate the safety of Bignay fruit extracts using chronic toxicity test.

The Bignay fruits analyzed by headspace GC–MS identified 16 compounds (Table 5). The major components of Bignay fruits were typical esters and aldehydes compounds, which may provide a distinct character with respect to flavor and aroma (Table 5). Some compounds are the simplest straight-chain unsaturated aldehyde of interest for perfumes and food flavors. Our findings serve as the basis in determining the possible benefits of Bignay for management of disease and commercial development. Further studies are required to isolate the active compounds of the extract as well as to clarify the exact mechanism of action of Bignay in various disorders.

We also measured qualitatively the secondary metabolites in Bignay fruit extracts. Phytochemical investigation showed the presence of various phytochemical constituents. Table 6 summarizes the secondary metabolites identified in extracted Bignay fruits, which are in agreement with those reported, by Luchai [21] and Islary [15]. Some of the identified metabolites have been demonstrated to hold pharmaceutical and ecological significance [45]. Our results showed high range of secondary metabolites, which are a characteristic feature of plants, are essential and can protect plants against a wide variety of microorganisms (viruses, bacteria, fungi) and herbivores (arthropods, vertebrates) and give plants characteristics such as color [45].

It is important to note some limitations of our findings. Only OECD 423, an acute oral toxicity test in mice, was used to evaluate the safety of extracted Bignay fruits. DNA barcoding [23] was not performed to identify the plant specimen but instead, accredited taxonomist (National Museum, Division of Botany, Philippines) conducted the authentication of plant specimen. Mice rather than rats or zebrafish was used as a model to conduct the acute toxicity test. It should also be noted that we used only ethanol for extraction as this solvent can dissolve both polar and non-polar constituents [46]. The results from the animal model of acute toxicity assessment do not automatically guarantee successful human and therapeutic interventions using such extract [47]. Although this study provides baseline information regarding its safety, it does not also identify the exact compound that may explain its medicinal role. Further studies on chronic toxicity (OECD 452) on *Antidesma bunius* (L.) Spreng (Bignay) fruits are also important to establish potential adverse effects, if any, of this valuable Bignay fruits.

We chose GC–MS because it is one of the best, fastest and precise methods to detect several compounds, including alcohols, long chain hydrocarbons, organic acids, steroids, esters, aldehydes, and amino acid and required a small amount or volume/weight of samples [48]. HPLC uses solvent mixtures and gradients, which is crucial part of HPLC and troubleshooting for better separation is often complex [48,49]. GC uses a pure inert gas as the mobile phase and its purpose is to carry molecules through the column and less complex. Furthermore, a previous study has reported that there are no significant differences between the values obtained with the two methods used [48,49]. We anticipated that the biological activities of Bignay fruits will provide positive results and will open an innovative area of investigation of individual components and their pharmacological potency.

## 5 Conclusion

This research offers important baseline information on the toxicity profile of Bignay fruits, as well as the first demonstration that harvested Bignay fruits are evidently safe when administered orally to mice at the maximum dose, 2000 mg/kg. Because no adverse effects were found in the hematology, biochemistry, or histopathology evaluations, the current study's findings suggest that Bignay taken orally could have a beneficial effect. More research on chronic toxicity tests is required before conducting clinical trials to ensure that *Antidesma bunius* (L.) Spreng berries [Bignay] do not pose a health risk to humans. Due to a lack of knowledge on mutagenicity and genotoxicity [50,51], more research is required before Bignay can be used as a beneficial product for health and wellness. Furthermore, the potency of secondary metabolites, the quantity consumed and the duration of exposure are contributory factors that need added examinations.

## Authors statement

We are submitting the newly revised manuscript for the captioned TOXREP-D-20-00211: “Acute Oral Toxicity Assessment of Ethanolic Extracts of *Antidesma bunius* (L.) Spreng Fruits in Mice *in Vivo*”, for your perusal. We are grateful that our manuscript will have a second review once the comments are fully addressed.

We thank the Reviewers for their kind comments of our work. The reviewers have made several suggestions for the betterment of our manuscript. Here, we have addressed the comments of the Reviewers and included the necessary changes in this newly revised manuscript.

## Authorship contribution statement

Conceptualization: MNMM.

Funding acquisition: MNMM, UAT.

Performed the assays: MNMM, JILR.

Methodology: MNMM, JILR.

Analyzed the data: MNMM, KW, UAT.

Writing - original draft: MNMM.

Writing - review and editing: MNMM, UAT, KW, JILR.

## Declaration of Competing Interest

The authors report no declarations of interest.
