# Supplementary material for: Acute oral toxicity assessment of ethanolic extracts of Antidesma bunius (L.) Spreng fruits in mice
Source: Toxicol Rep. 2021 Jun 17;8:1289–99. doi: 10.1016/j.toxrep.2021.06.010 (PMC8246092; doi:10.1016/j.toxrep.2021.06.010)
Supplement: Supplementary file 1 [file mmc1.docx]

**Supplemental data**

**SI Table 1.** Previous studies on characterization and evaluation of Bignay

| **AUTHOR** | **TITLE/JOURNAL** | **RESULTS** | |
| --- | --- | --- | --- |
| Butkhup L, Samappito S (2008) | An analysis on flavonoids contents in Mao Luang fruits of fifteen cultivars (Antidesma bunius), grown in northeast Thailand. Pakistan Journal of Biological Science 11:996-1002 | 15 Mao Luang cultivars contained three different kinds of flavonoids, i.e., catechin, procyanidin B1 and procyanidin B2. | |
| Chowtivannakul P, Srichaikul B, Talubmook C (2016). | Hypoglycemic and Hypolipidemic Effects of Seed Extract from Antidesma bunius (L.) Spreng in Streptozotocin-induced Diabetic Rats. Pakistan Journal of Biological Science 19:211-218. | The ethanol seed extract from A. bunius possesses hypoglycemic and hypolipidemic effects, likely due to its antioxidant and insulin secretion activities. | |
| [Walid Hamdy El-Tantawy](https://www.tandfonline.com/author/El-Tantawy%2C+Walid+Hamdy) et al. (2015) | I[nvestigation of antidiabetic action of *Antidesma bunius* extract in type 1 diabetes](https://www.tandfonline.com/doi/full/10.3109/13813455.2015.1038278?src=recsys). Archives of Physiology and Biochemistry  Volume 121, Issue 3 | A. bunius extract possess anti-diabetic activity, through the enhancement of hepatic glycogen storage and regeneration of the islet of Langerhans. | |
| Luchai, B., et al.  (2008) | [Analysis of Anthocyanin, Flavonoids, and Phenolic Acids in Tropical Bignay Berries](https://www.tandfonline.com/doi/full/10.1080/15538360802365913?src=recsys)  International Journal of Fruit Science  Volume 8, Issue 1-2 | The results suggest that methanolic extracts of bignay berries exhibit a potential for use as natural antioxidants. | |
| Luchai, B. and Supachai, S. (2011) | Changes in physicochemical properties, polyphenol compounds and antiradical activity during development and ripening of Maolung (Antidesma bunius L.Spreng) fruits. J.Fruit and Ornamental Plant Res., vol19(1): 85-99. | Maoluang possess the highest antioxidants and antiradical activity. | |
| Udomkasemsab A., Ngamlerst C., Adisakwattana P., Aroonnual A., Tungtrongchitr R., Prangthip P. (2018). | Maoberry (Antidesma bunius) ameliorates oxidative stress and inflammation in cardiac tissues of rats fed a high-fat diet.  BMC Complementary and Alternative Medicine 18:344. | Maoberry extract has remarkable effects on preventing progression of cardiac tissue deterioration at least through lowering oxidative stress and inflammation in rats. | |
| - [Ornnicha Krongyut](https://www.semanticscholar.org/author/Ornnicha-Krongyut/1420457634), [K. Sutthanut](https://www.semanticscholar.org/author/K.-Sutthanut/10158112) (2019) | [Phenolic Profile, Antioxidant Activity, and Anti-obesogenic Bioactivity of Mao Luang Fruits (Antidesma bunius L.)](https://www.semanticscholar.org/paper/Phenolic-Profile%2C-Antioxidant-Activity%2C-and-of-Mao-Krongyut-Sutthanut/943b44f02bef5d5d5d5dd8cb1af6f845675e7e7e)  Molecules, 24(22), 4109 | A potential anti-obesity agent contributed by inhibitory effects on lipase enzyme and anti-differentiation and -adipogenesis in adipocytes | |
| - [Islam](https://www.semanticscholar.org/author/M.-Islam/48189301), M. ,(2018) | [Phytochemical screening and evaluation of antioxidant and thrombolytic activities of methanolic extract of Antidesma bunius L. (Family Euphorbiaceae) leaves](https://www.semanticscholar.org/paper/Phytochemical-screening-and-evaluation-of-and-of-of-Islam-Biswas/6d4e249cbed50d6051e7ab6c0cae8012b51416ef)  International Journal of Unani and Integrative Medicine 2018; 2(3): 32-38 | The methanolic extract of leaves of *A. bunius* exhibited thrombolytic activity  and antioxidant property. | |
| - [Ibrahim](https://www.semanticscholar.org/author/T.-Ibrahim/5355544), T., et al. - (2019) | [Chemical composition and antimicrobial and cytotoxic activities of Antidesma bunius L.](https://www.semanticscholar.org/paper/Chemical-composition-and-antimicrobial-and-of-L.-Ibrahim-Dib/5469d5c13c91073a16d1da157d5f78113ba7ec5e)  Pakistan journal of pharmaceutical sciences 32(1):153-163 | Antimicrobial activity and cytotoxic effect of the plant could be attributed to its content of phenolic acids, flavan-3-ols and/or proanthocyanidins. | |
| [Hamidu](https://www.semanticscholar.org/author/La-Hamidu/95715498), L., [Ahmad](https://www.semanticscholar.org/author/Aktsar-Roskiana-Ahmad/145247652), A.R. [, Najib](https://www.semanticscholar.org/author/A.-Najib/72943071), A. (2018) | [Qualitative and Quantitative Test of Total Flavonoid Buni Fruit (Antidesma bunius (L.) Spreng) with UV-Vis Spectrophotometry Method](https://www.semanticscholar.org/paper/Qualitative-and-Quantitative-Test-of-Total-Buni-Hamidu-Ahmad/e02ec4239069530a8b2637e7046a790569f77ee1)  Pharmacognosy Journal. 10,1,60-63. | The result shows that the flavonoid content higher in the *n*-Hexane extract is 10.72 %, then ethyl acetate extract is 7.9 % and 3.56 % ethanol extract was counted to or as a Rutin. | |
| - [Zaman](https://www.semanticscholar.org/author/S.-Zaman/20618375), S., et al. (2018) | [Evaluation of Cytotoxicity and Antibacterial Activities of Methanolic Extract of Antidesma bunius (Linn.) (Family Euphorbiaceae) Leaf.](https://www.semanticscholar.org/paper/Evaluation-of-Cytotoxicity-and-Antibacterial-of-of-Zaman-Islam/27084c5b71c9da65a36f239f81ce8bf4a677db33) [[Journal of Advances in Medical and Pharmaceutical Sciences](https://www.semanticscholar.org/paper/Evaluation-of-Cytotoxicity-and-Antibacterial-of-of-Zaman-Islam/27084c5b71c9da65a36f239f81ce8bf4a677db33)](https://www.researchgate.net/journal/2394-1111_Journal_of_Advances_in_Medical_and_Pharmaceutical_Sciences)[16(2):1-7](https://www.semanticscholar.org/paper/Evaluation-of-Cytotoxicity-and-Antibacterial-of-of-Zaman-Islam/27084c5b71c9da65a36f239f81ce8bf4a677db33) | highest inhibition against microbial growth especially against E.coli  DH5∝ by having a zone of inhibition  highest inhibition against microbial growth especially against E.coli  DH5∝ by having a zone of inhibition  highest inhibition against microbial growth especially against E.coli DH5∝ by having a zone of inhibition; A. bunius leaves possess good cytotoxic activity | |
| [Pongnaratorn](https://www.semanticscholar.org/author/Panicha-Pongnaratorn/11773800), P. (2017) | [In vitro Antimicrobial activity of Antidesma bunius extracts on oral pathogenic bacteria](https://www.semanticscholar.org/paper/In-vitro-Antimicrobial-activity-of-Antidesma-bunius-Pongnaratorn/798cfe733c326468d4cb9741a03db303ccce4195)  [Thai Journal of Pharmaceutical Sciences](https://www.researchgate.net/journal/0125-4685_Thai_Journal_of_Pharmaceutical_Sciences) 41(4):144-149 | Possesses antibacterial property | |
| - [Chaikham](https://www.semanticscholar.org/author/P.-Chaikham/5947973), P. (2015) | [Comparison of high hydrostatic pressure and thermal processing on physicochemical and antioxidant properties of Maoberry (Antidesma thwaitesianum Müell. Arg.) juice.](https://www.semanticscholar.org/paper/Comparison-of-high-hydrostatic-pressure-and-thermal-Chaikham/bcb4e7062cb72a6302920ebe61f023a44fd75ca9)  International Food Research Journal, 22(5), 1993-2001. | Anti-oxidant property | |
| - [Sushma Khomdram Shyamananda Arambam](https://www.semanticscholar.org/author/Sushma-Khomdram-Shyamananda-Arambam/114012680), [Sharmistha Barthakur,et al.](https://www.semanticscholar.org/author/Sharmistha-Barthakur-Guruaribam-Shantibala-Devi/114427127) (2017) | [Biochemical, Nutritional Profiling and Optimization of an Efficient Nucleic Acid Isolation Protocol from Recalcitrant Tissue of Wild Edible Fruit Antidesma bunius L. Spreng.](https://www.semanticscholar.org/paper/Biochemical%2C-Nutritional-Profiling-and-Optimization-Arambam-Devi/77749f18ebce323df482a1a7441145fc51eecd2f)  Int.J.Curr.Microbiol.App.Sci . 6(4): 253-264 | Correlation  analysis showed significant relationship between ascorbic acid content and antioxidant  oxidant activity. | |
| - [Puspitasari](https://www.semanticscholar.org/author/E.-Puspitasari/83802566), E. (2009) | [Cytotoxicity Effect of Methanolic Extract of Buni’s Fruits (Antidesma bunius (L) Spreng) against Hela Cells](https://www.semanticscholar.org/paper/Cytotoxicity-Effect-of-Methanolic-Extract-of-Buni%E2%80%99s-Puspitasari/e888668fbd9c8631ec8979a63c869615d8061ba5)  Jurnal ILMU DASAR, [S.l.], v. 10, n. 2, p. 181-185; ISSN 2442-5613. | | Tested for its biological activity using BST (Brine Shrimp Lethality Test). Result showed toxic effect of methanolic extract of fruit. |
| Mwangomo T.D., Moshi J.M., Magadul J.J. (2012) | Thananant H, Satnako N. Antimicrobial activity of Mao berry  extract against Staphylococcus aureus and Propionibacterium  acnes. RSU National Research Conference; 2015. p. 184-91  Mwangomo TD, Moshi JM, Magadul JJ. Antimicrobial activity  and phytochemical screening of Antidesma venosum root and  stem bark ethanolic extracts. Int J Res Phytochem Pharmacol  2012;2:90-5.  Mwangomo TD, Moshi JM, Magadul JJ. Antimicrobial activity  and phytochemical screening of Antidesma venosum root and  stem bark ethanolic extracts. Int J Res Phytochem Pharmacol  2012;2:90-5.  Mwangomo TD, Moshi JM, Magadul JJ. Antimicrobial activity  and phytochemical screening of Antidesma venosum root and  stem bark ethanolic extracts. Int J Res Phytochem Pharmacol  2012;2:90-5.  Antimicrobial activity and phytochemical screening of Antidesma venosum root and stem bark ethanolic extracts. Int J Res Phytochem Pharmacol 2012;2:90-5. | | Anti-microbial property and phytochemical screening of Bignay |

**SI Table 2.** Mean Daily Feed and Water Intake of Treated Male Mice. There were no differences among treated animals. (n=5 mice). Bignay Fruit Extract (BFE).

| Treatment Groups | Daily Feed Intake (g) | Daily Water Intake (mL) |
| --- | --- | --- |
| Distilled water | 3.65 ± 0.05 | 3.80 ± 0.02 |
| 500 mg/kg BFE | 3.65 ± 0.04 | 3.82 ± 0.03 |
| 1000 mg/kg BFE | 3.66 ± 0.45 | 3.80 ± 0.90 |
| 2000 mg/kg BFE | 3.65 ± 0.04 | 3.81 ± 0.02 |

**SI Table 3**. Mean Daily Feed and Water Intake of Treated Female Mice. There were no differences among treated animals (n=5 mice). Bignay Fruit Extract (BFE).

| Treatment Groups | Daily Feed Intake (g) | Daily Water Intake (mL) |
| --- | --- | --- |
| Distilled water | 3.65 ± 0.30 | 3.80 ± 0.02 |
| 500 mg/kg BFE | 3.68 ± 0.01 | 3.75 ± 0.03 |
| 1000 mg/kg BFE | 3.66 ± 0.03 | 3.78 ± 0.01 |
| 2000 mg/kg BFE | 3.69 ± 0.01 | 3.74 ± 0.37 |

**SI Table 4**. Mean weight (grams) of visceral organs of male mice treated with distilled water and increasing doses of Bignay in mg/kg (n=5 mice). Bignay Fruit Extract (BFE).

| Treatment Groups | Esophagus  Stomach | Small Intestine | Large Intestine | Liver | Right Kidney | Left Kidney |
| --- | --- | --- | --- | --- | --- | --- |
| Distilled Water | 0.35 ± 0.04 | 1.43 ± 0.19 | 0.49 ± 0.06 | 1.54 ± 0.12 | 0.19 ± 0.03 | 0.18 ± 0.01 |
| 500 mg/kg  BFE | 0.33 ± 0.06 | 1.34 ± 0.22 | 0.48 ± 0.03 | 1.42 ±0.10 | 0.19 ± 0.01 | 0.17 ± 0.01 |
| 1000 mg/kg BFE | 0.33 ± 0.04 | 1.31 ± 0.22 | 0.48 ± 0.03 | 1.42 ± 0.13 | 0.17 ± 0.01 | 0.17 ± 0.01 |
| 2000 mg/kg BFE | 0.34 ± 0.03 | 1.32 ± 0.07 | 0.49 ± 0.04 | 1.44 ± 0.11 | 0.18 ± 0.01 | 0.18 ± .02 |

**SI Table 5**. Mean weight (grams) of visceral organs of female mice treated with distilled water and increasing doses of Bignay in mg/kg (n=5 mice). Bignay Fruit Extract (BFE).

| Treatment Groups | Esophagus  Stomach | Small Intestine | Large Intestine | Liver | Right Kidney | Left Kidney |
| --- | --- | --- | --- | --- | --- | --- |
| Distilled Water | 0.37 ± 0.05 | 1.58 ± 0.23 | 0.49 ± 0.03 | 1.45 ± 0.12 | 0.18 ± 0.01 | 0.18 ± 0.01 |
| 500 mg/kg  BFE | 0.35 ± 0.04 | 1.53 ± 0.13 | 0.48 ± 0.04 | 1.41 ± 0.10 | 0.18 ± 0.01 | 0.17 ± 0.01 |
| 1000 mg/kg BFE | 0.36 ± 0.02 | 1.53 ± 0.10 | 0.48 ± 0.03 | 1.39 ± 0.14 | 0.17 ± 0.01 | 0.18 ± 0.01 |
| 2000 mg/kg BFE | 0.35 ± 0.02 | 1.53 ± 0.16 | 0.48 ± 0.02 | 1.44 ± 0.06 | 0.18 ± 0.01 | 0.17 ± 0.02 |
